# 157. Long-Acting Injectable HIV Treatment in Individuals with Adherence Challenges: Real-World Insights from Southern California

**DOI:** 10.1093/ofid/ofae631.043

**Published:** 2025-01-29

**Authors:** Elizabeth Hastie, Lucas Hill, laura Bamford, Thomas CS Martin

**Affiliations:** UCSD, San Diego, CA; University of California, San Diego, San Diego, California; UCSD, San Diego, CA; University of California San Diego, La Jolla, California

## Abstract

**Background:**

Long-acting injectable antiretroviral therapy with cabotegravir and rilpivirine (LAI CAB/RPV) showed superiority over daily oral ART among persons with HIV (PWH) with adherence challenges in the LATITUDE study. This trial included conditional economic incentives (CEI) and compensation, which may limit generalizability. We aimed to assess real-world outcomes of LAI CAB/RPV among a similar population in a Ryan White funded primary care HIV clinic.

**Methods:**

PWH with adherence challenges who initiated LAI CAB/RPV at the UCSD Owen Clinic were included. Adherence challenges were defined as at least one HIV viral load (VL) > 200 copies/mL in the previous 12-months, along with either poor response to oral ART (two HIV VLs > 200 copies/mL separated by ≥ 4-weeks in the prior 18-months despite being prescribed oral ART) or being lost to clinical follow-up (no contact with an HIV provider for ≥ 6-months and non-adherence to ART for ≥ 7-days). The primary outcome was discontinuation of LAI CAB/RPV or VL ≥ 200 copies/mL within 48-weeks of initiation. Secondary outcome was confirmed virologic failure with resistance-associated mutations (RAMs).

**Results:**

63 PWH fulfilled the inclusion criteria: 71% male, 27% female and 2% non-binary, with a median age of 43 years. 46% were white, 16% black, and 38% Hispanic. 41% reported current or prior injection drug use, 40% had active substance use, and 57% had a psychiatric diagnosis. At LAI CAB/RPV initiation, 43% had a VL ≥ 50 copies/mL and 11% had a VL > 10,000 copies/ml. 14 (22%) participants, including all those with a detectable VL at baseline, were prescribed an additional ART for at least part of the 48-weeks. Of the 50 participants with virologic data to 48-weeks, 10 (20%) discontinued LAI CAB/RPV: 4 (8%) had virologic failure (VL ≥ 200 copies/mL) with RAMs and 6 (12%) discontinued for other reasons including participant preference and one participant death.

**Conclusion:**

This observational, real-world study demonstrated that PWH with adherence challenges experienced similar outcomes to those in clinical trials with CEI (20% with VL ≥ 200 copies/mL or LAI CAB/RPV discontinuation vs 24.1% in LATITUDE at 48-weeks). Our study’s lower primary outcome rate may be due to a higher-risk population being referred to LATITUDE to take advantage of CEI and compensation.

**Disclosures:**

**Lucas Hill, PharmD, AAHIVP**, ViiV Healthcare: Honoraria **Thomas CS Martin, MD**, Gilead Sciences: Grant/Research Support

